# NoncoRNA: a database of experimentally supported non-coding RNAs and drug targets in cancer

**DOI:** 10.1186/s13045-020-00849-7

**Published:** 2020-02-28

**Authors:** Lulu Li, Pengfei Wu, Zhenyu Wang, Xiangqi Meng, Caijun Zha, Ziwei Li, Tengfei Qi, Yangong Zhang, Bo Han, Shupeng Li, Chuanlu Jiang, Zheng Zhao, Jinquan Cai

**Affiliations:** 1 Department of Neurosurgery, the Second Affiliated Hospital of Harbin Medical University, Neuroscience Institute, Heilongjiang Academy of Medical Sciences, Harbin, 150086 China; 2 Department of Laboratory Diagnosis, the Second Affiliated Hospital of Harbin Medical University, Harbin, 150086 China; 3 Beijing Neurosurgical Institute, Beijing, 100070 China

**Keywords:** Database, Non-coding RNA, Drug resistance, Human cancer

## Abstract

NoncoRNA (http://www.ncdtcdb.cn:8080/NoncoRNA/) is a manually curated database of experimentally supported non-coding RNAs (ncRNAs) and drug target associations that aim to potentially provide a high-quality data resource for exploring drug sensitivity/resistance-related ncRNAs in various human cancers. ncRNA are RNA molecular that do not encode proteins, but are involved in gene regulation and cellular functions in variety of human diseases, including neurodegenerative diseases and cancers. Here, we developed NoncoRNA which contained 8233 entries between 5568 ncRNAs and 154 drugs in 134 cancers. Each entry in the NoncoRNA contains detailed information on the ncRNAs, drugs, and cancers, the ncRNA expression pattern and experimental detection techniques, drug response and other targets, literature references, and other information. NoncoRNA offers a user-friendly, open access web interface to easily browse, search, and download data. NoncoRNA also provides a submission page for researchers to submit newly validated ncRNA-drug-cancer associations. NoncoRNA might serve as an immeasurable resource for understanding the roles of ncRNAs in cancer therapy.

To the Editor,

Cancer is an important cause of morbidity and mortality worldwide, in every world region, and irrespective of the level of human development [1]. In addition, the incidence and mortality of cancer have increased rapidly worldwide in recent years. The reasons are complex, but drug resistance is the primary cause of clinical treatment failure [2]. However, the mechanism of drug resistance to chemotherapeutic agents is not fully elucidated. Currently, more and more evidences have been proved that non-coding RNAs play critical roles in drug resistance [3–6]. ncRNAs including long non-coding RNAs (lncRNAs), microRNAs (miRNAs), circular RNAs (circRNAs), and PIWI-interacting RNAs (piRNAs) are suggested to be the potential promising therapeutic targets for overcoming drug resistance in the treatment of human cancers [7–10]. Due to their functional importance, ncRNAs are under intense study at present, and some databases have been built to describe ncRNAs’ functional characterization, such as ncDR, Lnc2Cancer, SM2miR, CircR2Disease, and piRBase. Current ncRNA research related to drug resistance is mainly focused on lncRNAs and miRNAs. However, few studies have reported the association between circRNAs and piRNAs. Therefore, it is urgent demand to establish a database for more comprehensive data coverage drug resistance and ncRNAs in human cancers.

To fill this gap, we introduced NoncoRNA, a manually curated database of experimentally supported non-coding RNA and drug target associations in cancers. The current version contains 8233 entries involved in 5568 ncRNAs and 154 drugs in 134 cancers (Table 1). In addition, we also summarized the key ncRNAs of most common 10 drugs and the cancers associated ncRNAs in Additional file 1: Table S1 and S2.

**Table 1:** Data summary in NoncoRNA database

Features	NoncoRNA
Entries	8233
LncRNAs	3599
miRNAs	1006
circRNAs	959
piRNAs	4
Cancer subtypes	134
Drugs	154
Target gene	721
Pathways	340
Articles	1615

To establish a high-quality database of ncRNAs, all the articles related to drug resistance and ncRNAs were manually extracted from publications (Fig. 1). (i) We searched all published studies in the PubMed database using the following combination of keywords: “long non-coding RNA or lncRNA and drug and cancer,” “microRNA or miRNA and drug and cancer,” “circRNA and drug and cancer,” and “piRNA or PIWI-interacting RNA and drug and cancer.” Besides, we also integrated relevant information from ncDR and Lnc2Cancer. Then, we manually retrieved entries related to ncRNAs, drugs, and cancers by reading abstracts. (ii) The abstract and the full text of selected articles were manually screened to extract drug resistance-related ncRNAs and their detailed annotation information, such as article’s basic information, ncRNAs’ basic information, drug basic information, and the relationship between ncRNAs and drugs. The relationship between ncRNAs and drugs in cancers were supported by experiment, such as qRT-PCR, southern-blot, and high-throughput experiment. Similarly, we have provided experimental data related to ncRNAs and drug resistance in our laboratory, such as lnc-ERC1-1:5, lnc-SCG3-3:3, and NR_028415. (iii) In order to share information among different databases, we unified the information by certain criteria, including the drug ID from DrugBank and ncRNA ID from Ensembl, miRBase, circBase, piRBase, and so on. Then Disease Ontology and DrugBank were used to respectively unify cancer and drug annotations.

**Fig. 1:**
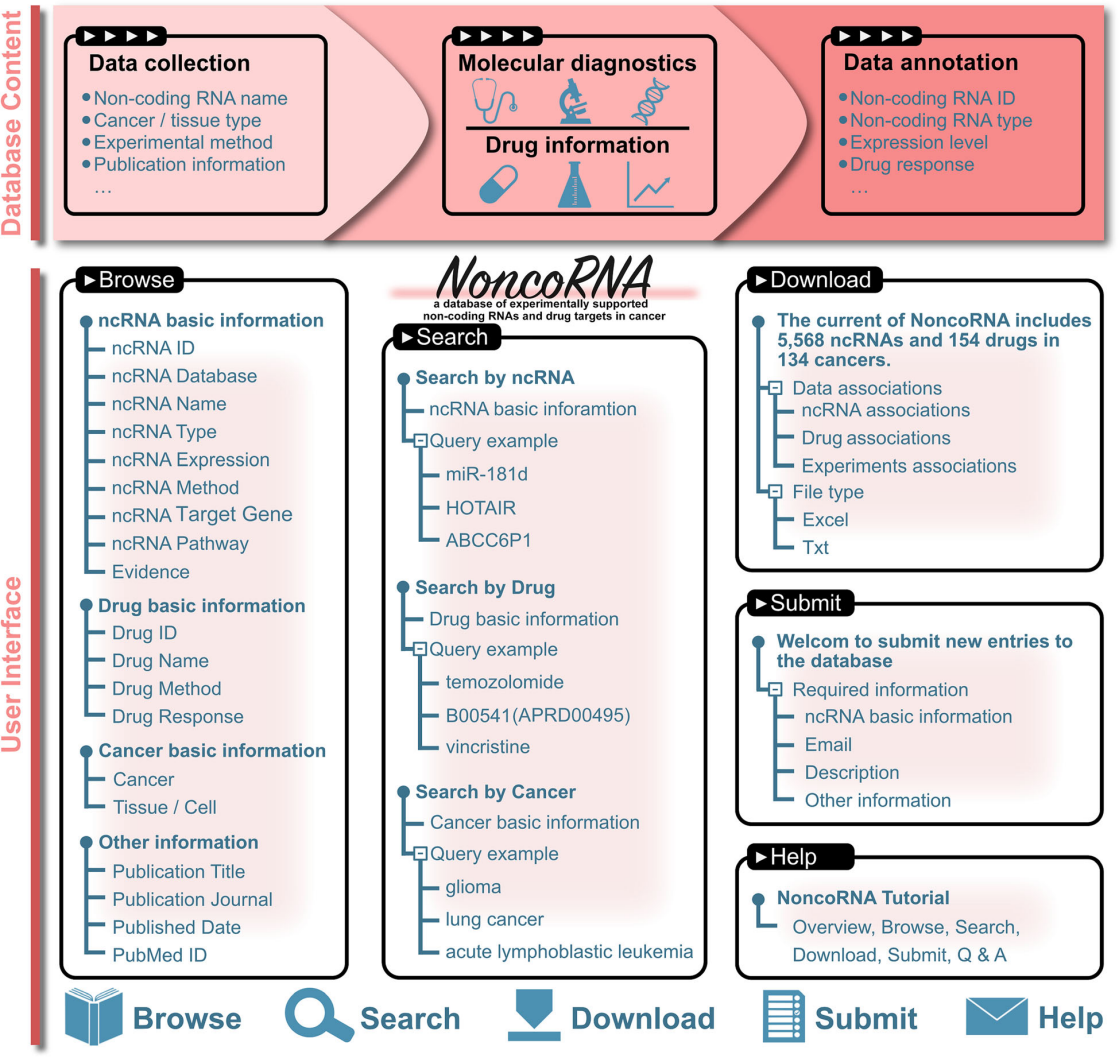
The workflow of the construction of NoncoRNA database

NoncoRNA offers a user-friendly, open access web interface to easily browse, search, and download data. Figure 1 shows the schematic workflow of NoncoRNA database. In the “search” page, users can search all entries in three ways: by ncRNAs, by drugs, or by cancers. In the “browse” page, users can browse by clicking “ncRNAs”, “drugs”, or “cancer type” according to their purpose, the information would be listed as leaf nodes. In addition, the NoncoRNA also provides “quick search” function in the home page and search interface, such as miR-200, glioma, and temozolomide. Through clicking “more”, you can obtain detailed information. Figure 1 shows the more details, the entry contains detailed information on the ncRNAs, drugs, and cancers, the ncRNA expression pattern and experimental detection techniques, drug response, target gene, pathways, literature references, and other information. In addition, all data in the database can be obtained by the function of download. The NoncoRNA provides two formats of the downloadable file in TXT and Excel formats, respectively. The submit function enables communication between users and websites. Users enable to submit novel experimentally supported ncRNA-drug-cancer association. The help function can teach users how to use NoncoRNA. In addition, the web proved some query examples, it also helps users better understand how to use NoncoRNA.

In conclusion, NoncoRNA may serve as an immeasurable source for drug resistance research in human cancer. Compared with other databases, the NoncoRNA includes 3 distinctive features: (i) first providing the relationship between circRNAs, piRNAs, and drug resistance in cancers; (ii) 3294 lncRNAs were provided from our laboratory database by microarray analysis, which has not been studied in the field of gliomas; (iii) exhibiting some relationship between ncRNAs and drug resistance in cancer. Meanwhile, we will update and improve the database every 2 months. So we can use this relationship to develop the function of ncRNA in different cancers and drugs, and even to predict the new function in other cancers and drugs. Anyway, NoncoRNA provides a reliable database platform for a wide range of scientific researchers.

## Supplementary information

**Additional file 1: Table S1.** Summary of the top 5 ncRNAs with references for most common 10 drugs. **Table S2.** Summary of cancers associated with ncRNAs.

## Data Availability

All data obtained and/or analyzed in this study were available from the NoncoRNA (http://www.ncdtcdb.cn:8080/NoncoRNA/).

## Abbreviations

circRNA: Circular RNA
lncRNA: Long non-coding RNA
miRNA: MicroRNA
ncRNA: Non-coding RNA
piRNA: PIWI-interacting RNA
qRT-PCR: Quantitative real time polymerase chain reaction
