# Supplementary material for: NoncoRNA: a database of experimentally supported non-coding RNAs and drug targets in cancer
Source: J Hematol Oncol. 2020 Feb 28;13:15. doi: 10.1186/s13045-020-00849-7 (PMC7048090; doi:10.1186/s13045-020-00849-7)
Supplement: Supplementary file 1 — Additional file 1: Table S1. Summary of the top 5 ncRNAs with references for most common 10 drugs. Table S2. Summary of cancers associated with ncRNAs. [file 13045_2020_849_MOESM1_ESM.docx]

**Table S1 Summary of the top 5 ncRNAs with references for most common 10 drugs.**

| **ncRNA Name** | **ncRNA Type** | **ncRNA Expression** | **Drug Name** | **Drug Response** | **Cancer Name** | **Target Gene** | **Reference (PMID)** |
| --- | --- | --- | --- | --- | --- | --- | --- |
| MALAT1 | lncRNA | ↑ | Temozolomide | ↓ | glioblastoma | ZEB1 | 28668966 |
| miR-125b | miRNA | ↑ | Temozolomide | ↓ | glioblastoma | BAK1 | 24643683 |
| miR-181b | miRNA | ↑ | Temozolomide | ↑ | glioma | MEK1 | 23645289 |
| miR-203 | miRNA | ↑ | Temozolomide | ↑ | glioma | E2F3 | 25515700 |
| miR-497 | miRNA | ↑ | Temozolomide | ↓ | glioma | PDCD4 | 25080009 |
| miR-21 | miRNA | ↑ | Cisplatin | ↓ | ovarian cancer | PTEN | 28789414 |
| miR-203 | miRNA | ↑ | Cisplatin | ↑ | lung non-small cell carcinoma | DKK1 | 28350100 |
| miR-200b | miRNA | ↓ | Cisplatin | ↓ | tongue cancer | BMI1 | 21725369 |
| miR-199a-3p | miRNA | ↑ | Cisplatin | ↑ | breast cancer | TFAM | 28126676 |
| miR-503 | miRNA | ↓ | Cisplatin | ↓ | lung non-small cell carcinoma | BCL2 | 23856992 |
| miR-218 | miRNA | ↑ | Oxaliplatin | ↑ | stomach cancer | SMO | 26261515 |
| miR-20a | miRNA | ↓ | Oxaliplatin | ↑ | colorectal adenocarcinoma | BNIP2 | 21242194 |
| miR-1915 | miRNA | ↑ | Oxaliplatin | ↑ | colorectal cancer | NFIX | 26695693 |
| miR-141 | miRNA | ↑ | Oxaliplatin | ↑ | colorectal cancer | Cyclin D2 | 28112364 |
| miR-139-5p | miRNA | ↑ | Oxaliplatin | ↑ | colorectal cancer | BCL2 | 27244080 |
| miR-21 | miRNA | ↑ | Doxorubicin | ↓ | bladder cancer | PTEN | 21468550 |
| miR-125b | miRNA | ↑ | Doxorubicin | ↓ | acute promyelocytic leukemia | BAK1 | 21880154 |
| miR-181b | miRNA | ↑ | Doxorubicin | ↓ | breast cancer | BIM | 26572075 |
| miR-101 | miRNA | ↑ | Doxorubicin | ↑ | hepatocellular carcinoma | EZH2 | 24211739 |
| miR-200c | miRNA | ↓ | Doxorubicin | ↓ | breast cancer | MDR1 | 22101791 |
| miR-497 | miRNA | ↑ | Fluorouracil | ↑ | colorectal cancer | IGF1R | 22710713 |
| miR-125b | miRNA | ↑ | Fluorouracil | ↓ | breast cancer | E2F3 | 22523546 |
| miR-122 | miRNA | ↑ | Fluorouracil | ↑ | hepatoblastoma | BCL2 | 22312705 |
| miR-145 | miRNA | ↓ | Fluorouracil | ↓ | stomach cancer | CD44 | 28428713 |
| miR-101 | miRNA | ↑ | Fluorouracil | ↑ | hepatocellular carcinoma | EZH2 | 24211739 |
| miR-200c | miRNA | ↑ | Paclitaxel | ↑ | ovarian cancer | TUBB3 | 23074172 |
| miR-21 | miRNA | ↑ | Paclitaxel | ↓ | ovarian cancer | APAF1 | 27021436 |
| miR-34a | miRNA | ↑ | Paclitaxel | ↑ | glioma | PD-L1 | 28721584 |
| miR-107 | miRNA | ↑ | Paclitaxel | ↑ | breast cancer | TPD52 | 31206033 |
| miR-130a | miRNA | ↓ | Paclitaxel | ↓ | prostate cancer | SLAIN1 | 26074357 |
| miR-21 | miRNA | ↑ | Gemcitabine | ↓ | breast cancer | PTEN | 26666820 |
| miR-181b | miRNA | ↑ | Gemcitabine | ↓ | pancreatic cancer | CYLD | 24075517 |
| miR-143 | miRNA | ↑ | Gemcitabine | ↑ | bladder cancer | IGF-1R | 28123579 |
| miR-330 | miRNA | ↓ | Gemcitabine | ↑ | lung cancer | DCK | 22132977 |
| miR-1246 | miRNA | ↑ | Gemcitabine | ↓ | pancreatic cancer | CCNG2 | 25117811 |
| miR-497 | miRNA | ↑ | Vincristine | ↑ | stomach cancer | BCL2 | 21258880 |
| miR-129-5p | miRNA | ↑ | Vincristine | ↑ | breast cancer | SOX4 | 27831649 |
| miR-126 | miRNA | ↑ | Vincristine | ↑ | lung non-small cell carcinoma | VEGFA | 22510476 |
| miR-15b | miRNA | ↑ | Vincristine | ↑ | stomach cancer | BCL2 | 18449891 |
| miR-181b | miRNA | ↑ | Vincristine | ↑ | lung cancer | BCL2 | 20162574 |
| miR-34a-5p | miRNA | ↑ | Etoposide | ↓ | osteosarcoma | CD117 | 27056900 |
| miR-181b | miRNA | ↑ | Etoposide | ↑ | lung cancer | BCL2 | 20162574 |
| miR-424 | miRNA | ↑ | Etoposide | ↓ | melanoma | PDCD4 | 24967963 |
| miR-15b | miRNA | ↑ | Etoposide | ↑ | stomach cancer | BCL2 | 18449891 |
| miR-20a-5p | miRNA | ↓ | Etoposide | ↓ | osteosarcoma | SDC2 | 29118673 |
| miR-125a-3p | miRNA | ↑ | Docetaxel | ↑ | breast cancer | BRCA1 | 27693788 |
| miR-141 | miRNA | ↓ | Docetaxel | ↑ | lung non-small cell carcinoma | EIF4E | 27840955 |
| miR-27b | miRNA | ↑ | Docetaxel | ↑ | prostate cancer | ZEB1 | 29102917 |
| miR-34a | miRNA | ↑ | Docetaxel | ↑ | prostate cancer | ZEB1 | 29102917 |
| miR-21 | miRNA | ↑ | Docetaxel | ↓ | prostate cancer | PDCD4 | 20581857 |

**Table S2 Summary of cancers associated with ncRNAs**

| **Cancer Name** | **lncRNA** | **miRNA** | **circRNA** | **piRNA** | **Total** |
| --- | --- | --- | --- | --- | --- |
| acute lymphocytic leukemia | 3 | 7 | 0 | 0 | 10 |
| acute myeloid leukemia | 4 | 24 | 1 | 0 | 29 |
| acute myeloid leukemia (except M3) | 0 | 1 | 0 | 0 | 1 |
| acute promyelocytic leukemia | 0 | 1 | 0 | 0 | 1 |
| acute T cell leukemia | 0 | 1 | 0 | 0 | 1 |
| adrenocortical carcinoma | 0 | 1 | 0 | 0 | 1 |
| anaplastic thyroid cancer | 1 | 1 | 0 | 0 | 2 |
| anaplastic thyroid carcinoma | 0 | 2 | 0 | 0 | 2 |
| bladder cancer | 6 | 44 | 1 | 0 | 51 |
| bladder transitional cell carcinoma | 2 | 0 | 0 | 0 | 2 |
| bladder urothelial carcinoma | 0 | 1 | 0 | 0 | 1 |
| breast adenocarcinoma | 0 | 4 | 0 | 0 | 4 |
| breast cancer | 56 | 373 | 20 | 2 | 451 |
| breast carcinoma | 0 | 2 | 0 | 0 | 2 |
| breast tumor | 0 | 5 | 0 | 0 | 5 |
| cancer | 1 | 23 | 0 | 0 | 24 |
| cervical cancer | 10 | 20 | 0 | 0 | 30 |
| cervical squamous cell carcinoma | 0 | 1 | 0 | 0 | 1 |
| cholangiocarcinoma | 0 | 9 | 0 | 0 | 9 |
| chondrosarcoma | 0 | 3 | 0 | 0 | 3 |
| chordoma | 0 | 6 | 0 | 0 | 6 |
| chronic lymphocytic leukemia | 0 | 4 | 0 | 0 | 4 |
| chronic myelocytic leukemia | 0 | 1 | 0 | 0 | 1 |
| chronic myelogenous leukemia | 0 | 1 | 0 | 0 | 1 |
| chronic myeloid leukemia | 5 | 37 | 3 | 0 | 45 |
| clear cell renal cell carcinoma | 0 | 2 | 0 | 0 | 2 |
| colon cancer | 47 | 104 | 0 | 0 | 151 |
| colon carcinoma | 0 | 1 | 0 | 0 | 1 |
| colorectal adenocarcinoma | 0 | 4 | 0 | 2 | 6 |
| colorectal cancer | 50 | 112 | 55 | 0 | 217 |
| colorectal carcinoma | 0 | 10 | 0 | 0 | 10 |
| colorectal tumors | 0 | 1 | 0 | 0 | 1 |
| cutaneous T-cell lymphomas | 0 | 1 | 0 | 0 | 1 |
| diffuse large B-cell lymphoma | 0 | 57 | 0 | 0 | 57 |
| EML4-ALK positive NSCLC | 0 | 2 | 0 | 0 | 2 |
| endometrial cancer | 1 | 6 | 0 | 0 | 7 |
| endometrial carcinoma | 2 | 4 | 0 | 0 | 6 |
| endometrioid ovarian cancer | 0 | 1 | 0 | 0 | 1 |
| epidermoid carcinoma | 0 | 5 | 0 | 0 | 5 |
| epithelial ovarian cancer | 2 | 14 | 0 | 0 | 16 |
| epithelial ovarian carcinoma | 0 | 2 | 0 | 0 | 2 |
| esophageal adenocarcinoma | 0 | 5 | 0 | 0 | 5 |
| esophageal cancer | 2 | 70 | 0 | 0 | 72 |
| esophageal carcinoma | 0 | 2 | 0 | 0 | 2 |
| esophageal squamous cell carcinoma | 6 | 25 | 0 | 0 | 31 |
| esophagus adenocarcinoma | 0 | 1 | 0 | 0 | 1 |
| ewing sarcoma | 0 | 5 | 0 | 0 | 5 |
| gallbladder cancer | 1 | 5 | 0 | 0 | 6 |
| gastric adenocarcinoma | 0 | 47 | 0 | 0 | 47 |
| gastric carcinoma | 1 | 3 | 0 | 0 | 4 |
| gastrointestinal stromal tumor | 1 | 49 | 0 | 0 | 50 |
| germ cell tumor | 0 | 72 | 0 | 0 | 72 |
| glioblastoma | 3,291 | 52 | 0 | 0 | 3,343 |
| glioblastoma multiforme | 0 | 1 | 0 | 0 | 1 |
| glioma | 8 | 27 | 1 | 0 | 36 |
| head and neck cancer | 0 | 2 | 0 | 0 | 2 |
| head and neck squamous cell carcinoma | 0 | 42 | 0 | 0 | 42 |
| hepatic carcinoma | 0 | 1 | 0 | 0 | 1 |
| hepatitis B virus-associated hepatocellular carcinoma | 0 | 1 | 0 | 0 | 1 |
| hepatoblastoma | 0 | 1 | 0 | 0 | 1 |
| hepatocarcinoma | 0 | 3 | 0 | 0 | 3 |
| hepatocellular cancer | 3 | 1 | 0 | 0 | 4 |
| hepatocellular carcinoma | 151 | 367 | 0 | 0 | 518 |
| Her2-receptor positive breast cancer | 0 | 1 | 0 | 0 | 1 |
| human negroid cervix epitheloid carcinoma | 0 | 1 | 0 | 0 | 1 |
| kidney cancer | 0 | 9 | 0 | 0 | 9 |
| KRAS Mutant Tumor | 0 | 2 | 0 | 0 | 2 |
| laryngeal cancer | 0 | 7 | 0 | 0 | 7 |
| laryngeal carcinoma | 1 | 1 | 0 | 0 | 2 |
| laryngeal squamous cell carcinoma | 1 | 0 | 0 | 0 | 1 |
| leukemia | 2 | 28 | 0 | 0 | 30 |
| liver cancer | 7 | 98 | 0 | 0 | 105 |
| lung adenocarcinoma | 27 | 49 | 0 | 0 | 76 |
| lung cancer | 7 | 76 | 0 | 0 | 83 |
| lung carcinoma | 0 | 2 | 0 | 0 | 2 |
| lung non-small cell carcinoma | 48 | 141 | 52 | 0 | 241 |
| lung small cell carcinoma | 7 | 52 | 0 | 0 | 59 |
| lung squamous cell carcinoma | 36 | 2 | 0 | 1 | 39 |
| lymphocytic leukemia | 0 | 2 | 0 | 0 | 2 |
| lymphoid malignancy | 0 | 1 | 0 | 0 | 1 |
| malignant mesothelioma | 0 | 1 | 0 | 0 | 1 |
| malignant pleural mesothelioma | 1 | 4 | 0 | 0 | 5 |
| mantle cell lymphoma | 5 | 3 | 0 | 0 | 8 |
| medulloblastoma | 0 | 5 | 0 | 0 | 5 |
| melanoma | 1 | 24 | 0 | 0 | 25 |
| multiple myeloma | 3 | 18 | 0 | 0 | 21 |
| myeloid leukemia | 0 | 1 | 0 | 0 | 1 |
| nasopharyngeal cancer | 0 | 1 | 0 | 0 | 1 |
| nasopharyngeal carcinoma | 4 | 14 | 0 | 0 | 18 |
| nasopharynx cancer | 2 | 1 | 0 | 0 | 3 |
| neuroblastoma | 0 | 23 | 0 | 1 | 24 |
| oesophageal adenocarcinoma | 0 | 2 | 0 | 0 | 2 |
| oesophageal cancer | 1 | 4 | 0 | 0 | 5 |
| oral cancer | 0 | 2 | 0 | 0 | 2 |
| oral squamous cell carcinoma | 4 | 7 | 1 | 0 | 12 |
| oral tongue squamous cell cancer | 0 | 1 | 0 | 0 | 1 |
| osteosarcoma | 43 | 90 | 74 | 0 | 207 |
| ovarian cancer | 61 | 217 | 0 | 0 | 278 |
| ovarian carcinoma | 0 | 8 | 0 | 0 | 8 |
| ovarian papillary serous carcinoma | 0 | 1 | 0 | 0 | 1 |
| ovarian serous carcinoma | 0 | 8 | 0 | 0 | 8 |
| ovarian squamous cell carcinoma | 4 | 0 | 0 | 0 | 4 |
| paediatric acute lymphoblastic leukaemia | 0 | 1 | 0 | 0 | 1 |
| pancreas cancer | 0 | 1 | 0 | 0 | 1 |
| pancreatic adenocarcinoma | 0 | 1 | 0 | 0 | 1 |
| pancreatic cancer | 32 | 120 | 0 | 0 | 152 |
| pancreatic carcinoma | 0 | 5 | 0 | 0 | 5 |
| pancreatic ductal adenocarcinoma | 3 | 8 | 2 | 0 | 13 |
| papillary thyroid cancer | 1 | 0 | 0 | 0 | 1 |
| papillary thyroid carcinoma | 1 | 1 | 0 | 0 | 2 |
| pediatric acute lymphoblastic leukemia | 0 | 5 | 0 | 0 | 5 |
| pediatric intracranial nongerminomatous malignant germ cell tumors | 0 | 1 | 0 | 0 | 1 |
| peripheral T-cell lymphoma | 0 | 5 | 0 | 0 | 5 |
| Ph(+) leukemia | 0 | 2 | 0 | 0 | 2 |
| Precursor T-lymphoblastic lymphoma/leukemia | 2 | 0 | 0 | 0 | 2 |
| primitive neuroectodermal tumor | 0 | 3 | 0 | 0 | 3 |
| prostate cancer | 23 | 46 | 826 | 0 | 895 |
| renal carcinoma | 0 | 4 | 0 | 0 | 4 |
| renal cell carcinoma | 0 | 10 | 0 | 0 | 10 |
| retinoblastoma | 0 | 4 | 0 | 0 | 4 |
| retinoblastoma cancer | 0 | 5 | 0 | 0 | 5 |
| sarcoma | 2 | 0 | 0 | 0 | 2 |
| seminoma | 1 | 1 | 0 | 0 | 2 |
| squamous carcinoma | 0 | 3 | 0 | 0 | 3 |
| squamous cell carcinoma | 2 | 3 | 0 | 0 | 5 |
| stomach cancer | 34 | 255 | 1 | 0 | 290 |
| synovial sarcoma | 0 | 2 | 0 | 0 | 2 |
| testicular embryonal carcinoma | 0 | 1 | 0 | 0 | 1 |
| thyroid cancer | 0 | 61 | 0 | 0 | 61 |
| tongue cancer | 0 | 4 | 0 | 0 | 4 |
| tongue squamous cell carcinoma | 1 | 26 | 0 | 0 | 27 |
| triple-receptor negative breast cancer | 0 | 2 | 0 | 0 | 2 |
| urothelial carcinoma | 0 | 1 | 0 | 0 | 1 |
| uterine sarcoma | 0 | 1 | 0 | 0 | 1 |
